# Hijacking anaerobic metabolism to restore antibiotic efficacy in *Pseudomonas aeruginosa*

**DOI:** 10.1128/aem.01425-25

**Published:** 2025-09-29

**Authors:** Zealon Gentry-Lear, Celine Lopez Padilla, Melanie A. Spero

**Affiliations:** 1 Institute of Molecular Biology, University of Oregon 3265, Eugene, Oregon, USA; Indiana University Bloomington, Bloomington, Indiana, USA

**Keywords:** antibiotic tolerance, anaerobic metabolism, synergy, antibiotic potentiation, chlorate, nitrate respiration

## Abstract

Bacterial pathogens regularly encounter oxygen (O_2_) limitation at sites of infection in the human body. Many pathogens exhibit antibiotic recalcitrance under low O_2_ conditions, spotlighting the need for new therapeutics that are effective in O_2_-limited environments. We demonstrate that several classes of antibiotics display limited toxicity against hypoxic cultures of the opportunistic pathogen, *Pseudomonas aeruginosa*, where the pathogen exhibits antibiotic tolerance. In O_2_-limited host environments, many pathogens employ nitrate respiration, marking this anaerobic bacterial metabolism as an excellent drug target. Chlorate is a nitrate analog that hijacks nitrate respiration to kill *P. aeruginosa*. Chlorate acts as a prodrug: nitrate reductase reduces chlorate, thereby forming the toxic oxidizing agent, chlorite. Chlorate treatment is most toxic to *P. aeruginosa* under anoxia and displays limited-to-no toxicity under hypoxic or oxic conditions. Thus, neither chlorate nor antibiotics alone efficiently kill O_2_-limited *P. aeruginosa*. Excitingly, combined chlorate-antibiotic treatment showed that chlorate addition potentiates all tested classes of antibiotics, often eradicating hypoxic *P. aeruginosa* populations to below detection. Chlorate addition reduced the lethal dose of ceftazidime by >100-fold, further highlighting chlorate’s capacity to potentiate antibiotic treatment. Unlike chlorate, we found that most antibiotics do not synergize with different classes of drugs, except for colistin. Given that combination therapy is a promising strategy for combating antibiotic treatment failure, future studies should continue exploring chlorate’s therapeutic potential, including examining the mechanisms of chlorate-antibiotic synergy. Our work points to the critical relationship between bacterial physiology and drug efficacy and highlights anaerobic metabolism as a promising drug target.

**IMPORTANCE:**

Many antibiotics are less effective at killing pathogens under oxygen (O_2_)-limited conditions. Pathogens frequently encounter O_2_ limitation within host environments, which helps explain why antibiotic therapies often fail to resolve chronic infections. We are investigating the relationship between O_2_ availability and drug efficacy in the opportunistic pathogen, *Pseudomonas aeruginosa*. In agreement with prior work, we demonstrate that *P. aeruginosa* exhibits antibiotic recalcitrance under hypoxic conditions. We also explore the use of a novel therapeutic, chlorate, which kills *P. aeruginosa* under O_2_-limited conditions when the pathogen utilizes anaerobic metabolism (nitrate respiration). Excitingly, we find that chlorate-antibiotic combinations are highly lethal to *P. aeruginosa* across a wide range of O_2_ availabilities similar to those the pathogen encounters during infection. Our work demonstrates that we can leverage our understanding of pathogen physiology to propose novel drug combinations that hijack anaerobic metabolism to overcome antibiotic treatment failure in O_2_-limited environments.

## INTRODUCTION

Bacterial pathogens routinely encounter hypoxic and anoxic microenvironments within the human body (1, 2). Oxygen (O_2_) limitation is particularly common at sites of infection, where O_2_ is locally depleted by both host cells and microbes through aerobic respiration, and also by immune cells, which rapidly consume local O_2_ through processes like the respiratory burst (3, 4). O_2_ limitation has profound effects on bacterial physiology, including that it causes many facultative anaerobes to grow slowly. Slow-growth states can have devastating, real-world consequences because many pathogens become highly tolerant to antibiotics under O_2_-limited, slow-growing conditions (5–7).

The relationship between environmental hypoxia and antibiotic treatment failure is thought to underpin different types of recalcitrant infections, including chronic wound and cystic fibrosis (CF) airway infections. In chronic wounds, which affect ~2% of the U.S. population (8), tissue hypoxia stems from insufficient blood supply as well as local O_2_ consumption by microbes and overactive immune cells (9, 10). Chronic wounds cannot heal while there is an active infection, yet antibiotic treatments often fail to resolve wound infections, leading to complications like limb amputation (11). The airways of people with CF are coated with a thick mucus, called sputum, that is largely hypoxic or anoxic due to immune cell activity (3, 12). Pathogens grow slowly in the CF airway environment (13–15), which likely contributes to the decades-long persistence of CF lung infections despite aggressive antibiotic regimens (16). Finally, biofilms are a hallmark of recalcitrant infections, and pathogens are known to form biofilms in chronic wound tissue, CF sputum, and other contexts (17–20). Biofilms are notoriously tolerant to high antibiotic concentrations because they harbor slow-growing, O_2_-limited populations (21, 22).

To overcome hypoxia-associated antibiotic treatment failure, we propose using drugs that target anaerobic bacterial metabolisms. Nitrate respiration is a widespread form of anaerobic energy metabolism that supports the growth or survival of many pathogens in hypoxic host environments (23, 24). During nitrate respiration, nitrate reductase reduces nitrate (NO_3_^−^) to nitrite (NO_2_^−^). Although there are several types of nitrate reductases, only the Nar enzyme directly contributes to energy conservation by coupling nitrate reduction to the formation of a proton motive force (25). There is strong evidence that Nar-mediated nitrate respiration supports pathogen survival or growth in the host. Enteric pathogens, including *Salmonella enterica* and *Escherichia coli*, use Nar to respire host-derived nitrate to boost their growth in the inflamed gut (26–30). In *Mycobacterium* spp., nitrate respiration supports persistence in different host models (31–33). *Brucella suis* also appears to use nitrate respiration to replicate in macrophages and hypoxic environments (34, 35), and *Burkholderia* spp. were shown to upregulate *nar* within the host environment (36) and when grown as a biofilm (37). Finally, we recently showed that the opportunistic pathogen *Pseudomonas aeruginosa* requires Nar to cause persistent chronic wound infections in diabetic mice (38). Taken together, nitrate respiration is a promising therapeutic target for killing pathogens in hypoxic or anoxic host environments.

**Fig 1:**
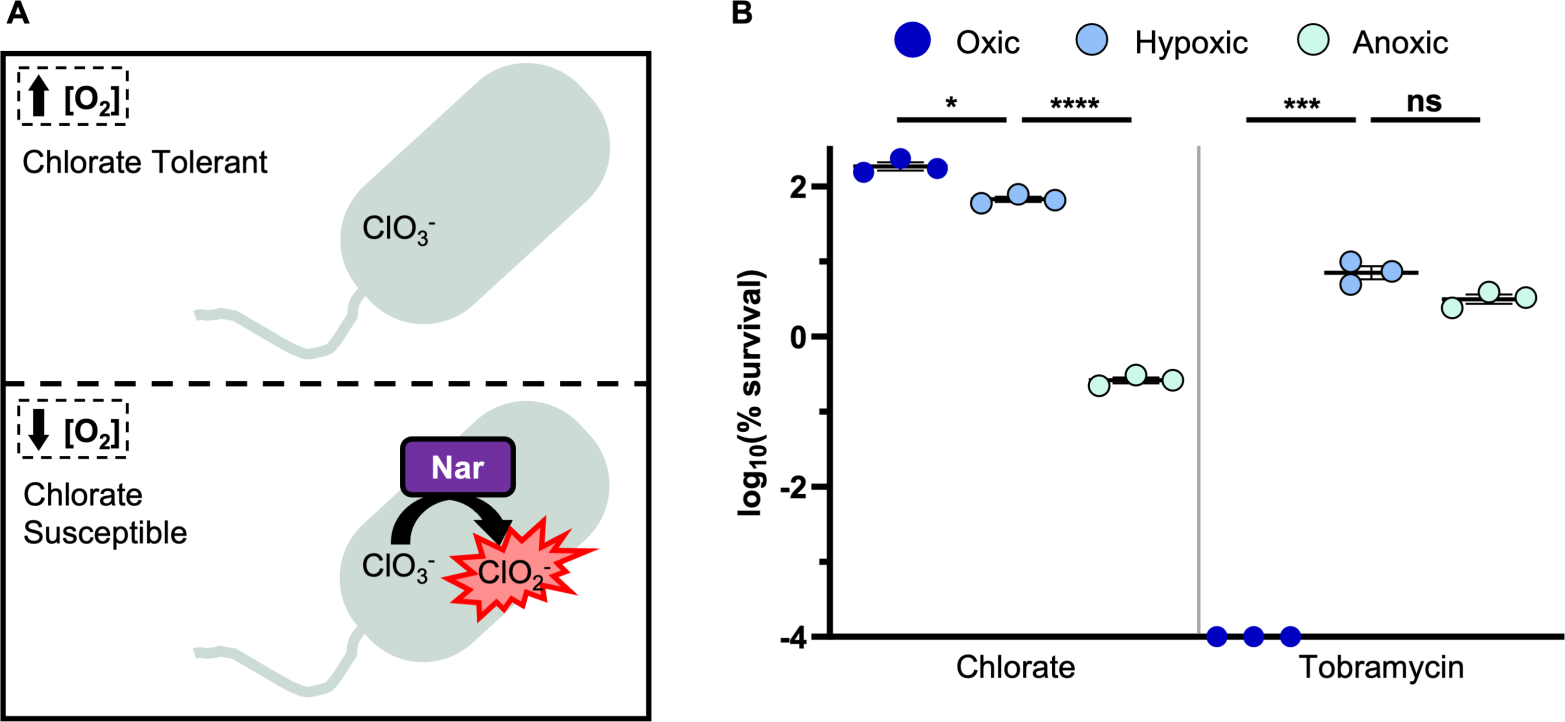
Chlorate hijacks the hypoxically induced Nar enzyme to kill anoxic, antibiotic-tolerant *P. aeruginosa*. (**A**) Chlorate is a prodrug: while chlorate itself is relatively nontoxic, it is reduced to toxic chlorite by the hypoxically induced nitrate reductase, Nar. (**B**) The log_10_(% survival) of wild type (WT) *P. aeruginosa* cultures treated with tobramycin or chlorate for 24 hours incubated under oxic, hypoxic, or anoxic conditions. Final drug concentrations were 31.25 µg/mL tobramycin and 10 mM chlorate. All data points on the x-axis are below the detection limit. Data show the means of three replicates, and error bars show SEM. Statistical significance was determined by one-way Welch’s analysis of variance (ANOVA) followed by Dunnett’s T3 post-hoc test for multiple comparisons; ns = not significant, * = *P* < 0.05, ** = *P* < 0.01, *** = *P* < 0.001, and **** = *P* < 0.0001.

It has long been known that the small molecule, chlorate, is toxic to bacteria when they use Nar-dependent nitrate respiration (39). Chlorate (ClO_3_^−^) is a nitrate analog that acts as a prodrug: chlorate itself is relatively nontoxic, but Nar can bind and reduce chlorate to generate chlorite (ClO_2_^−^), which is a toxic oxidizing agent (Fig. 1A) (40, 41). Mammals lack Nar, so it is unsurprising that chlorate shows low toxicity in mammals, with an estimated lethal oral dose of 20–35 g for humans (42, 43). Chlorate efficacy and toxicity have been best studied in animal husbandry, where livestock were orally dosed with chlorate (via drinking water or feed) with the goal of reducing fecal shedding of enteric pathogens, since many enteric bacteria utilize Nar. Orally dosed chlorate was shown to reduce fecal enteric counts in swine (44–50), poultry (51–56), sheep (57–61), and cows (62–64), without adverse health effects (41). Additionally, there is no evidence that chlorate’s toxic product, chlorite, accumulates during treatment. Studies using radiolabeled chlorate do not detect chlorite in tissues or excreta of food animals, instead demonstrating that chlorate is metabolized to chloride (65–69). In our own studies, we were also unable to detect chlorite in the supernatants of chlorate-reducing *P. aeruginosa* cultures (70). These findings suggest that chlorite, which is generated within the bacterial cytoplasm, interacts with cytoplasmic components (e.g., proteins) (71) and does not accumulate extracellularly to harm the host.

We previously showed that chlorate kills O_2_-limited, antibiotic-tolerant populations of *P. aeruginosa* biofilms via Nar activity (70). This was the first time demonstrating that chlorate can be used to kill antibiotic-recalcitrant bacteria, including those that form biofilms and exhibit antibiotic tolerance. In addition to our *in vitro* studies, we recently showed that topical chlorate treatment supports the healing of *P. aeruginosa*-infected chronic wounds in a diabetic mouse model, with no observed cytotoxicity (38). Finally, we expect that pathogens like *P. aeruginosa* are unlikely to develop chlorate resistance in the host environment. In prior work, we found that the primary mechanism of chlorate resistance in *P. aeruginosa* is to acquire Nar-inactivating mutations (71), yet *P. aeruginosa* requires Nar to persist in the chronic wound environment (38). As mentioned above, many pathogens are known or suspected to require Nar for infection (26–38), so strains that evolve chlorate resistance would likely exhibit severe fitness defects in the host.

Here, we continue to explore chlorate’s therapeutic potential by investigating its interactions with antibiotics across a range of O_2_ availabilities. Excitingly, we found that chlorate potentiates various classes of antibiotics to eliminate hypoxic, antibiotic recalcitrant populations of *P. aeruginosa*. We also evaluated the toxicity of different antibiotic-antibiotic combinations against O_2_-limited *P. aeruginosa*, finding that most antibiotics do not exhibit synergistic interactions across multiple drug classes. Our results demonstrate that combined chlorate-antibiotic treatment holds promise for combating antibiotic treatment failure in hypoxic host environments.

## RESULTS

### *P. aeruginosa* exhibits antibiotic recalcitrance under hypoxic conditions

Given the prevalence of hypoxia in host environments, we were interested in exploring the relationship between O_2_ availability and drug efficacy in *P. aeruginosa*. Prior work from ourselves and others has shown that some antibiotics are less effective at killing pathogens under O_2_-limited, slow-growth conditions (22, 70, 72). We investigated drug efficacy against *P. aeruginosa* cultures under a range of O_2_ availabilities. Briefly, *P. aeruginosa* was grown overnight under hypoxic conditions (static incubation) in LB supplemented with nitrate. Overnight cultures were pelleted and resuspended in fresh LB medium (lacking nitrate) at a high cell density (OD_500_ = 2). Cultures were incubated with or without drug(s) for 24 hours under the following conditions to achieve oxic, hypoxic, or anoxic environments: incubated with vigorous shaking (oxic conditions), statically (hypoxic conditions), or in an anaerobic glove box (anoxic conditions). Lastly, cells were plated to determine viable cell counts for quantifying percent survival.

The *nar* operon is upregulated in response to both O_2_ limitation and increased nitrate concentrations (73). Drug treatment assays were conducted in the absence of nitrate but across a range of O_2_ availabilities. Given that the Nar enzyme is more active under low O_2_ compared to high O_2_ conditions, where it reduces chlorate to generate toxic chlorite (Fig. 1A), it was unsurprising that chlorate treatment was nonlethal to oxic *P. aeruginosa* cultures but resulted in ~2.5-log killing under anoxic conditions (Fig. 1B). Interestingly, chlorate showed almost no toxicity against hypoxic *P. aeruginosa* cultures (0.2-log killing; Fig. 1B), suggesting that either Nar was inactive under these conditions or that the cell was able to defend itself from Nar-generated chlorite stress. We found that the antibiotic tobramycin was highly lethal to *P. aeruginosa* under oxic conditions (>6-log killing) but much less toxic to *P. aeruginosa* under hypoxic or anoxic conditions (~1.5-log killing; Fig. 1B). These results are consistent with prior work, demonstrating that O_2_ limitation causes *P. aeruginosa* to grow slowly and adopt an antibiotic-tolerant state (22, 70, 74, 75).

**Fig 2:**
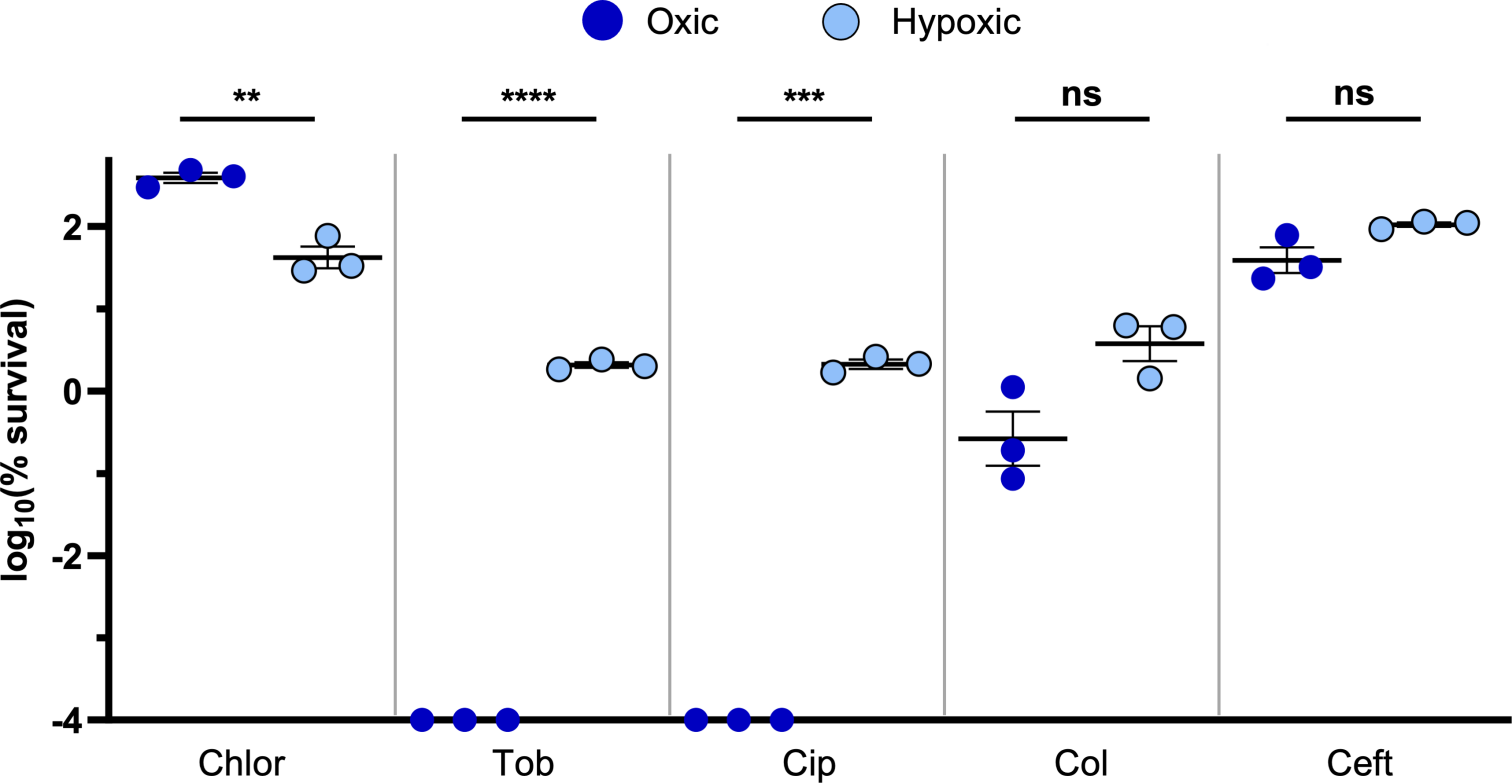
*P. aeruginosa* exhibits antibiotic recalcitrance under hypoxic conditions. The log_10_(% survival) of WT *P. aeruginosa* cultures treated with chlorate (Chlor), tobramycin (Tob), ciprofloxacin (Cip), colistin (Col), or ceftazidime (Ceft) for 24 hours incubated under hypoxic or oxic conditions. Final drug concentrations were 10 mM chlorate, 31.25 µg/mL tobramycin, 1 µg/mL ciprofloxacin, 15 µg/mL colistin, and 10 µg/mL ceftazidime. All data points on the x-axis are below the detection limit. Data show the means of three replicates, and error bars show SEM. Statistical significance was determined by two-tailed Welch’s *t*-tests; ns = not significant, * = *P* < 0.05, ** = *P* < 0.01, *** = *P* < 0.001, and **** = *P* < 0.0001.

We next aimed to determine whether other antibiotics exhibit O_2_-dependent toxicities, similar to tobramycin. We focused our studies on different classes of anti-pseudomonal antibiotics: aminoglycosides (tobramycin), fluoroquinolones (ciprofloxacin), beta-lactams (cephalosporins: ceftazidime), and polymyxins (colistin). We first determined the MIC for each drug, showing that *P. aeruginosa* UCBPP-PA14 is considered susceptible or intermediate for each antibiotic (Table S1). To test for O_2_-dependent toxicity, we incubated high-density cultures of *P. aeruginosa* with each drug under oxic or hypoxic conditions, using antibiotic concentrations that exceed the MIC and approximate those measured in patient samples (76, 77). As before, high-density *P. aeruginosa* cultures were incubated with or without antibiotic treatment under oxic or hypoxic conditions for 24 hours before plating cells to determine viability. Both tobramycin and ciprofloxacin were substantially more toxic to *P. aeruginosa* under oxic (>6-log killing) compared to hypoxic (~1.5-log killing; Fig. 2) conditions. However, colistin and ceftazidime were only marginally, but not statistically, more effective at killing oxic compared to hypoxic cultures (Fig. 2). Thus, *P. aeruginosa* exhibits recalcitrance to all tested antibiotics under hypoxic conditions, although hypoxia-induced antibiotic tolerance was most evident for tobramycin and ciprofloxacin.

### Chlorate potentiates different classes of antibiotics to eliminate hypoxic *P. aeruginosa* populations

Although all tested drug treatments showed modest-to-no toxicity against hypoxic cultures of *P. aeruginosa* (Fig. 2), we reasoned that each drug might impose sufficient stress on the cell such that combined chlorate-antibiotic treatment would efficiently kill hypoxic *P. aeruginosa* cultures. Here, we defined chlorate potentiation as occurring when chlorate addition resulted in ≥1.5-log killing more than the predicted additive amount of chlorate-antibiotic killing; the predicted additive amount of killing was calculated by summing the observed amount of killing for both single-drug treatments that comprise the drug combination.

**Fig 3:**
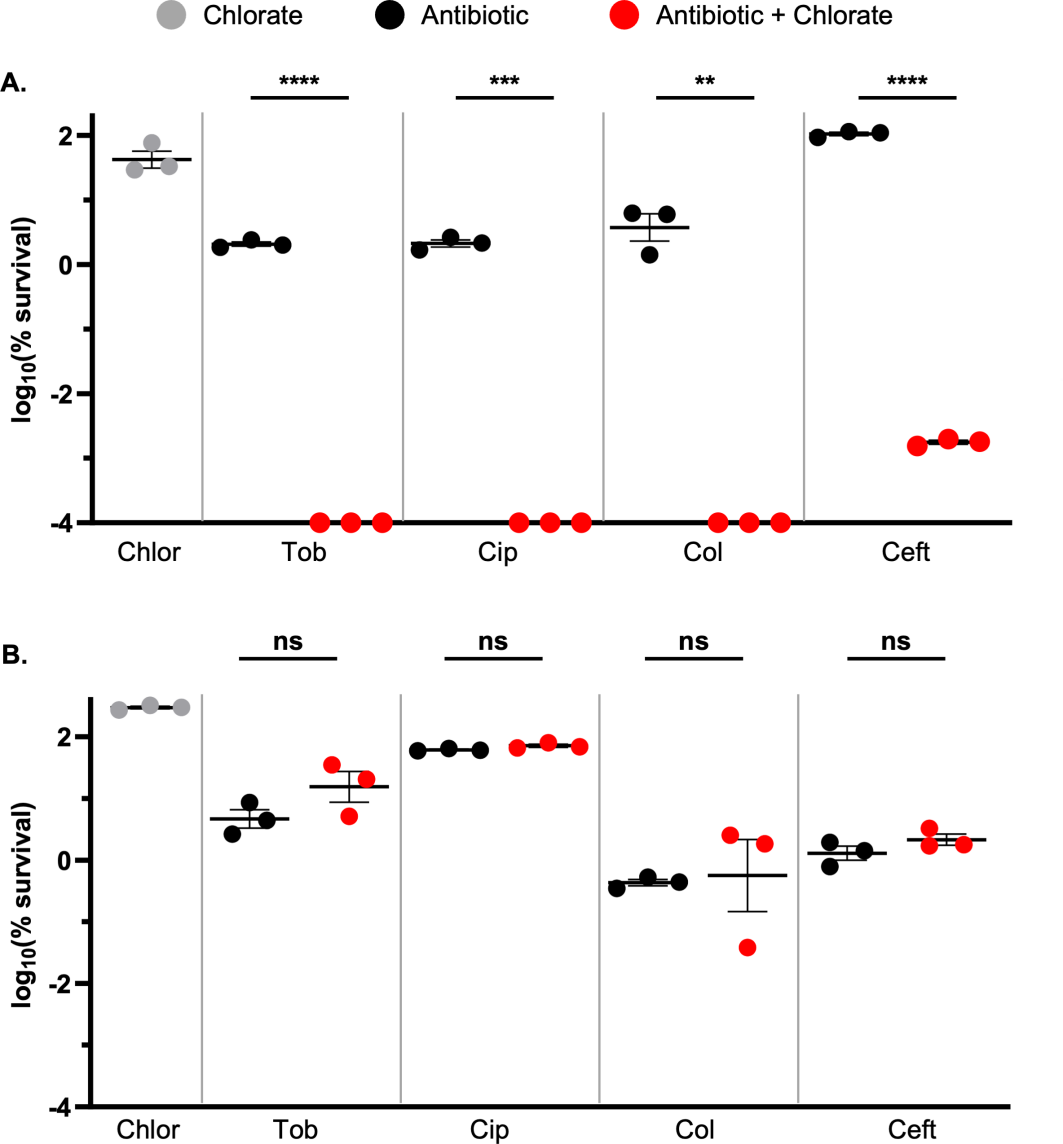
Chlorate potentiates different classes of antibiotics via Nar activity to kill hypoxic *P. aeruginosa* populations. The log_10_(% survival) of (**A**) WT or (**B**) ∆*nar P. aeruginosa* cultures treated with chlorate (Chlor), tobramycin (Tob), ciprofloxacin (Cip), colistin (Col), ceftazidime (Ceft), or each chlorate-antibiotic combination for 24 hours incubated under hypoxic conditions. Final drug concentrations were 10 mM chlorate, 31.25 µg/mL tobramycin, 1 µg/mL ciprofloxacin, 15 µg/mL colistin, and 10 µg/mL ceftazidime. Chlorate was defined as potentiating antibiotic treatment when the amount of observed killing in the chlorate-antibiotic combination was ≥1.5 log more than the predicted additive amount of killing, the latter of which was calculated by summing the observed log-killing values of chlorate-only and antibiotic-only treatments. All data points on the x-axis are below the detection limit. Data show the means of three replicates, and error bars show SEM. Statistical significance was determined by two-tailed Welch’s *t*-tests; ns = not significant, * = *P* < 0.05, ** = *P* < 0.01, *** = *P* < 0.001, and **** = *P* < 0.0001.

Indeed, we found that chlorate potentiates all tested antibiotics (Fig. 3A). Despite chlorate-only treatment resulting in little-to-no killing of hypoxic *P. aeruginosa* cultures, chlorate’s addition to each antibiotic treatment increased killing by more than four orders of magnitude for all tested classes of antibiotics (Fig. 3A). To confirm that chlorate reduction (i.e., chlorite generation) is required for antibiotic potentiation (as opposed to chlorate itself driving potentiation), we conducted similar experiments using hypoxic cultures of a *P. aeruginosa* ∆*nar* strain. As expected, potentiation was abolished in the ∆*nar* strain, where antibiotic-only and combined chlorate-antibiotic treatments resulted in the same level of killing (Fig. 3B).

**Fig 4:**
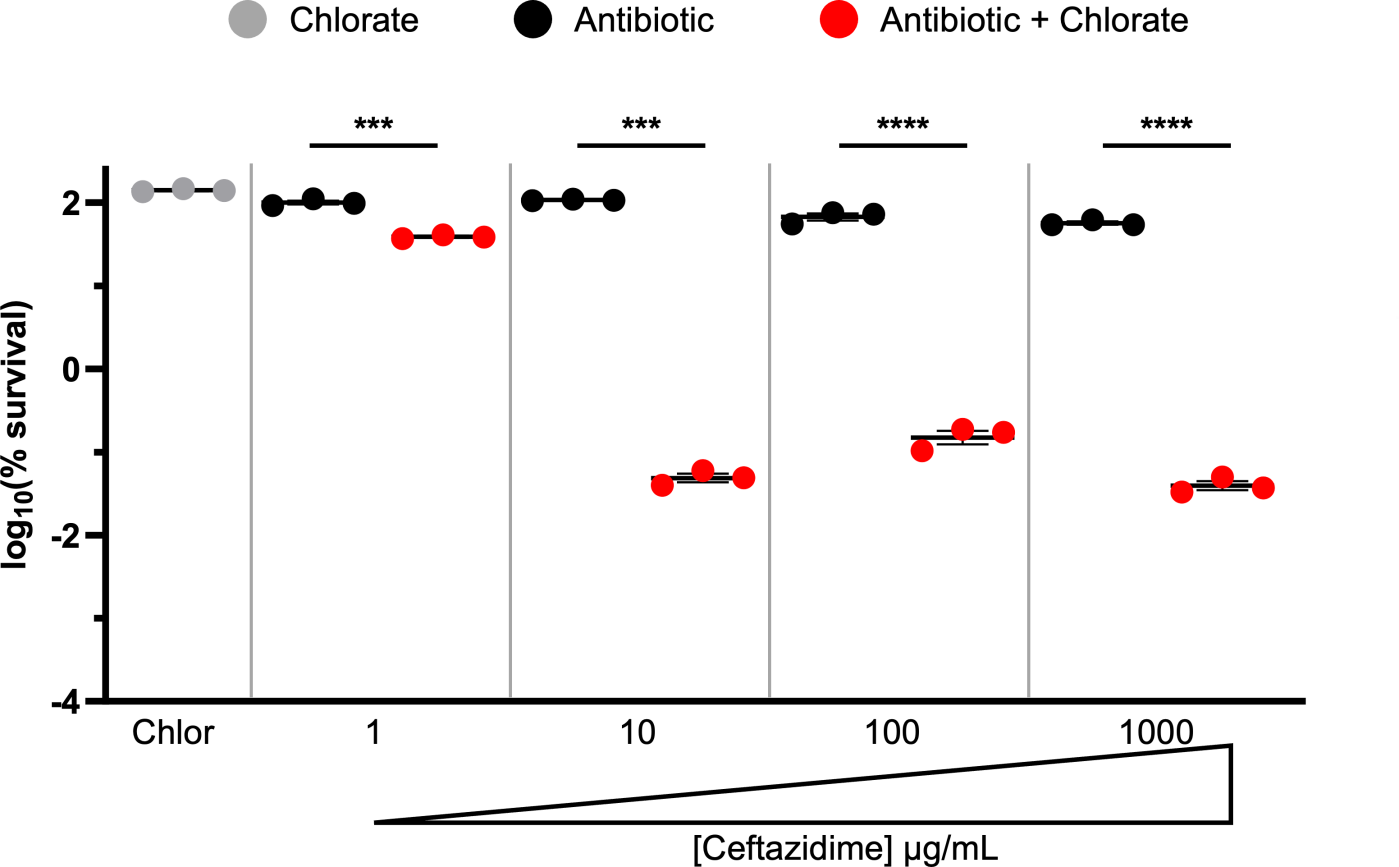
Chlorate addition substantially lowers the effective ceftazidime dose for killing hypoxic *P. aeruginosa*. The log_10_(% survival) of WT *P. aeruginosa* cultures treated with chlorate (Chlor), a range of ceftazidime (Ceft) concentrations, or chlorate-ceftazidime combinations for 24 hours incubated under hypoxic conditions. Final drug concentrations were 10 mM chlorate and 1, 10, 100, or 1,000 µg/mL ceftazidime. Chlorate was defined as potentiating antibiotic treatment when the amount of observed killing in the chlorate-antibiotic combination was ≥1.5-log more than the predicted additive amount of killing, the latter of which was calculated by summing the observed log-killing values of chlorate-only and antibiotic-only treatments. All data points on the x-axis are below the detection limit. Data show the means of three replicates, and error bars show SEM. Statistical significance was determined by two-tailed Welch’s *t*-tests; ns = not significant, * = *P* < 0.05, ** = *P* < 0.01, *** = *P* < 0.001, and **** =*P* < 0.0001.

We further examined the interaction between chlorate and ceftazidime, given that ceftazidime-only treatment resulted in no killing of hypoxic cultures of *P. aeruginosa* (Fig. 2), whereas *P. aeruginosa* was highly susceptible to combined chlorate-ceftazidime treatment (Fig. 3A). Our initial experiments used ceftazidime at a concentration of 10 µg/mL; however, we found that *P. aeruginosa* survives exposure to significantly higher concentrations (Fig. 4). By treating hypoxic cultures of *P. aeruginosa* for 24 hours with increasing ceftazidime concentrations, we saw that *P. aeruginosa* survives ceftazidime concentrations as high as 1,000 µg/mL, far exceeding concentrations that can be achieved in the body when this drug is administered to patients (76–79). Treating hypoxic cultures of *P. aeruginosa* with the chlorate-ceftazidime combination reduced the effective ceftazidime dose by >100-fold, since chlorate-ceftazidime is toxic to *P. aeruginosa* at a ceftazidime concentration of just 10 µg/mL (3.5-log killing; Fig. 4), which is well below concentrations achieved in patients. Thus, chlorate addition can significantly reduce the effective dose of an antibiotic under O_2_-limited conditions.

### Chlorate potentiates antibiotics across a range of O_2_ availabilities

**Fig 5:**
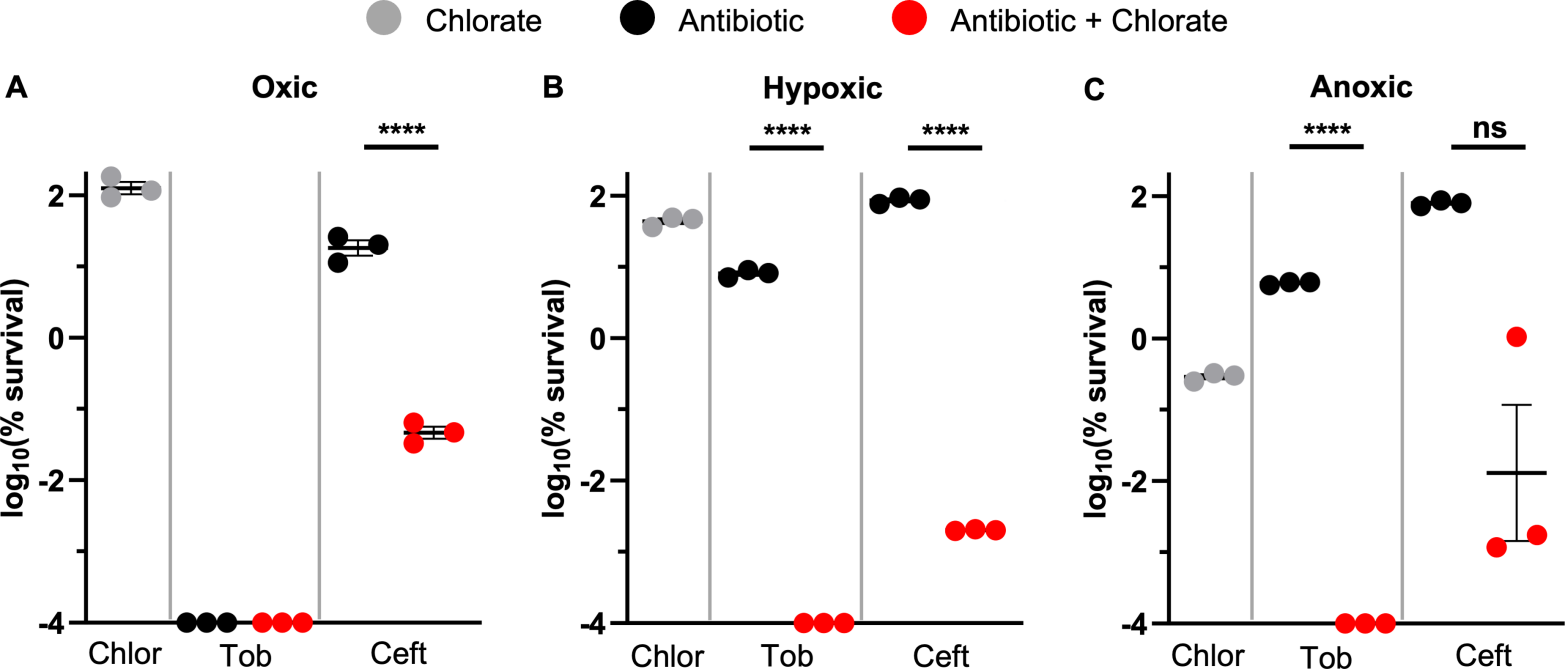
Chlorate-antibiotic potentiation is effective across a range of O_2_ availabilities. The log_10_(% survival) of WT *P. aeruginosa* cultures treated with chlorate (Chlor), tobramycin (Tob), ceftazidime (Ceft), or each chlorate-antibiotic combination for 24 hours incubated under (**A**) oxic, (**B**) hypoxic, or (**C**) anoxic conditions. Final drug concentrations were 10 mM chlorate, 31.25 µg/mL tobramycin, and 10 µg/mL ceftazidime. Chlorate was defined as potentiating antibiotic treatment when the amount of observed killing in the chlorate-antibiotic combination was ≥1.5-log more than the predicted additive amount of killing, the latter of which was calculated by summing the observed log-killing values of chlorate-only and antibiotic-only treatments. All data points on the x-axis are below the detection limit. Data show the means of three replicates, and error bars show SEM. Statistical significance was determined by two-tailed Welch’s *t*-tests; ns = not significant, * = *P* < 0.05, ** = *P* < 0.01, *** = *P* < 0.001, and **** =*P* < 0.0001.

Given that chlorate and some antibiotic treatments (tobramycin, ciprofloxacin) exhibit strong O_2_-dependent efficacy, we reasoned that chlorate-antibiotic potentiation might also vary across different O_2_ availabilities. To test this idea, we treated *P. aeruginosa* for 24 hours with different drug combinations under oxic, hypoxic, or anoxic conditions. We first tested the chlorate-tobramycin combination (Fig. 5). During single-drug treatments, chlorate and tobramycin exhibit opposing O_2_-dependent toxicities (i.e., chlorate and tobramycin are most effective under anoxic and oxic conditions, respectively). Tobramycin treatment eliminated *P. aeruginosa* to below our detection limit under oxic conditions, so we cannot determine whether chlorate potentiates tobramycin under these conditions (Fig. 5A). However, similar to hypoxic conditions (Fig. 5B), we found that combined chlorate-tobramycin treatment also kills anoxic cultures of *P. aeruginosa* to below our detection limit (Fig. 5C). Thus, it was promising to find that this drug combination maintains efficacy under anoxic conditions.

Next, we examined the chlorate-ceftazidime combination across different O_2_ availabilities. Unlike tobramycin, ceftazidime exhibits little to no toxicity under oxic conditions (0.4–0.7 log killing, Fig. 2 and 5A, respectively). Interestingly, chlorate-ceftazidime treatment showed similar levels of killing under oxic, hypoxic, and anoxic conditions (~3.5- to 4.5-log killing; Fig. 5). The oxic results were surprising and suggest that chlorate treatment induces sufficient cellular stress to potentiate ceftazidime even under more oxygenated conditions. Importantly, our use of the term oxic to describe experimental conditions is relative, since even a vigorously shaken high-density culture will experience O_2_ limitation; however, we also note that O_2_ concentrations are sufficiently different between oxic and hypoxic conditions to drive large changes in antibiotic efficacy for some drugs (e.g., tobramycin and ciprofloxacin; Fig. 2). To support the model that high-density *P. aeruginosa* cultures experience some O_2_ limitation even under shaking oxic conditions, we conducted similar experiments using the ∆*nar* strain. Indeed, we found that *P. aeruginosa* requires Nar for chlorate to enhance ceftazidime treatment under oxic conditions (Fig. S1), demonstrating that this organism utilizes the nitrate respiration pathway under these conditions. Overall, we demonstrate that Nar-mediated chlorate reduction enhances antibiotic efficacy across a range of O_2_ availabilities, as achieved by anoxic, static, or vigorous shaking conditions.

### Chlorate potentiates antibiotic therapy against *P. aeruginosa* clinical isolates

**Fig 6:**
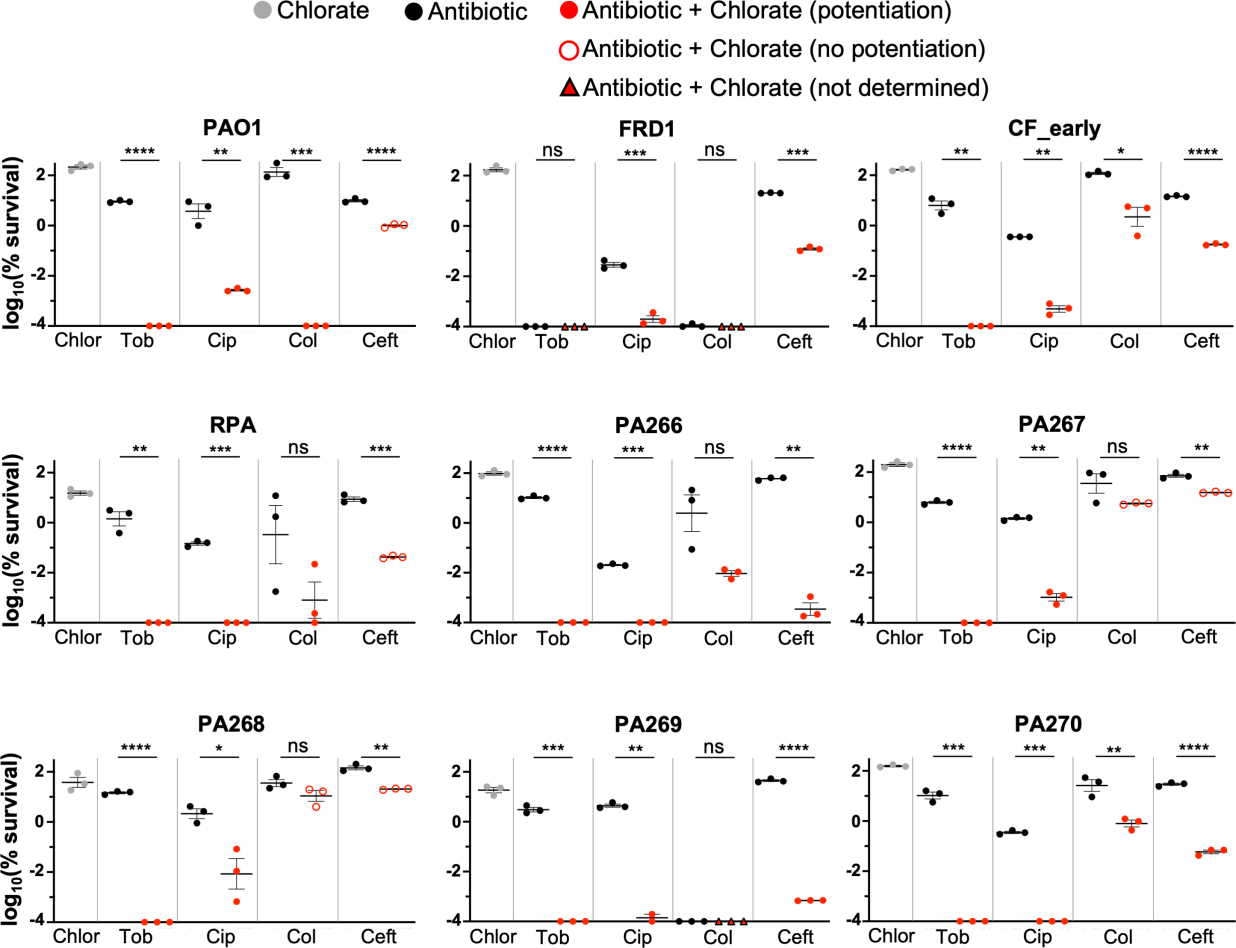
Chlorate enhances antibiotic efficacy against *P. aeruginosa* clinical isolates. The log_10_(% survival) of nine *P*. *aeruginosa* isolates treated with chlorate (Chlor), tobramycin (Tob), ciprofloxacin (Cip), colistin (Col), ceftazidime (Ceft), or each chlorate-antibiotic combination for 24 hours incubated under hypoxic conditions. Final drug concentrations were 10 mM chlorate, 31.25 µg/mL tobramycin, 1 µg/mL ciprofloxacin, 15 µg/mL colistin, and 10 µg/mL ceftazidime. Chlorate was defined as potentiating antibiotic treatment when the amount of observed killing in the chlorate-antibiotic combination was ≥1.5-log more than the predicted additive amount of killing, the latter of which was calculated by summing the observed log-killing values of chlorate-only and antibiotic-only treatments. Chlorate-potentiating and -nonpotentiating combinations are distinguished with solid red and outlined red data points, respectively. Chlorate potentiation could not be determined in instances where antibiotic-only treatment achieved substantial killing, indicated by triangle data points. All data points on the x-axis are below the detection limit. Data show the means of 2–3 replicates, and error bars show SEM. Statistical significance was determined by two-tailed Welch’s *t*-tests; ns = not significant, * = *P* < 0.05, ** = *P* < 0.01, *** = *P* < 0.001, and **** = *P* < 0.0001.

To this point, all experiments were conducted with the common laboratory strain, *P. aeruginosa* UCBPP-PA14. We next sought to test chlorate-antibiotic combinations against other *P. aeruginosa* isolates. We tested nine additional isolates, including another common laboratory strain (PAO1), a characterized isolate from a diabetic mouse model of chronic wounds (RPA [38]), and several mucoid and non-mucoid isolates collected from the sputum of people with CF, including well-characterized isolates like FRD1 (80) and CF_early (81) (see “Materials and Methods” for more detail). All *P. aeruginosa* isolates were incubated with or without drug(s) for 24 hours under hypoxic conditions before plating cultures to determine percent survival. Unsurprisingly, clinical isolates exhibited a range of sensitivities to antibiotic-only treatments. We were unable to determine chlorate potentiation in a small number of cases where antibiotic-only treatment killed the isolate to below our detection limit (e.g., isolate FRD1 was killed to below detection in tobramycin-only and colistin-only treatments, Fig. 6).

Excitingly, chlorate potentiates antibiotic efficacy in all isolates (Fig. 6). The chlorate-tobramycin combination was particularly lethal, reducing *P. aeruginosa* viability to below our detection limit (4- to 5-log more killing than tobramycin-only treatment) for all determinable isolates (eight of eight). Chlorate also potentiated ciprofloxacin treatment in all isolates (nine of nine). Chlorate addition potentiated colistin and ceftazidime killing in most determinable isolates (five of seven and five of nine, respectively). Although chlorate addition did not always meet our definition of potentiation (≥1.5-log more killing than the predicted additive amount of chlorate-antibiotic killing), combined chlorate-antibiotic treatment always resulted in increased killing compared to antibiotic-only treatments. Overall, chlorate-antibiotic combinations overcome antibiotic recalcitrance to kill various *P. aeruginosa* isolates under O_2_-limited conditions.

### Most antibiotics do not enhance killing in combination with other classes of antibiotics

**Fig 7:**
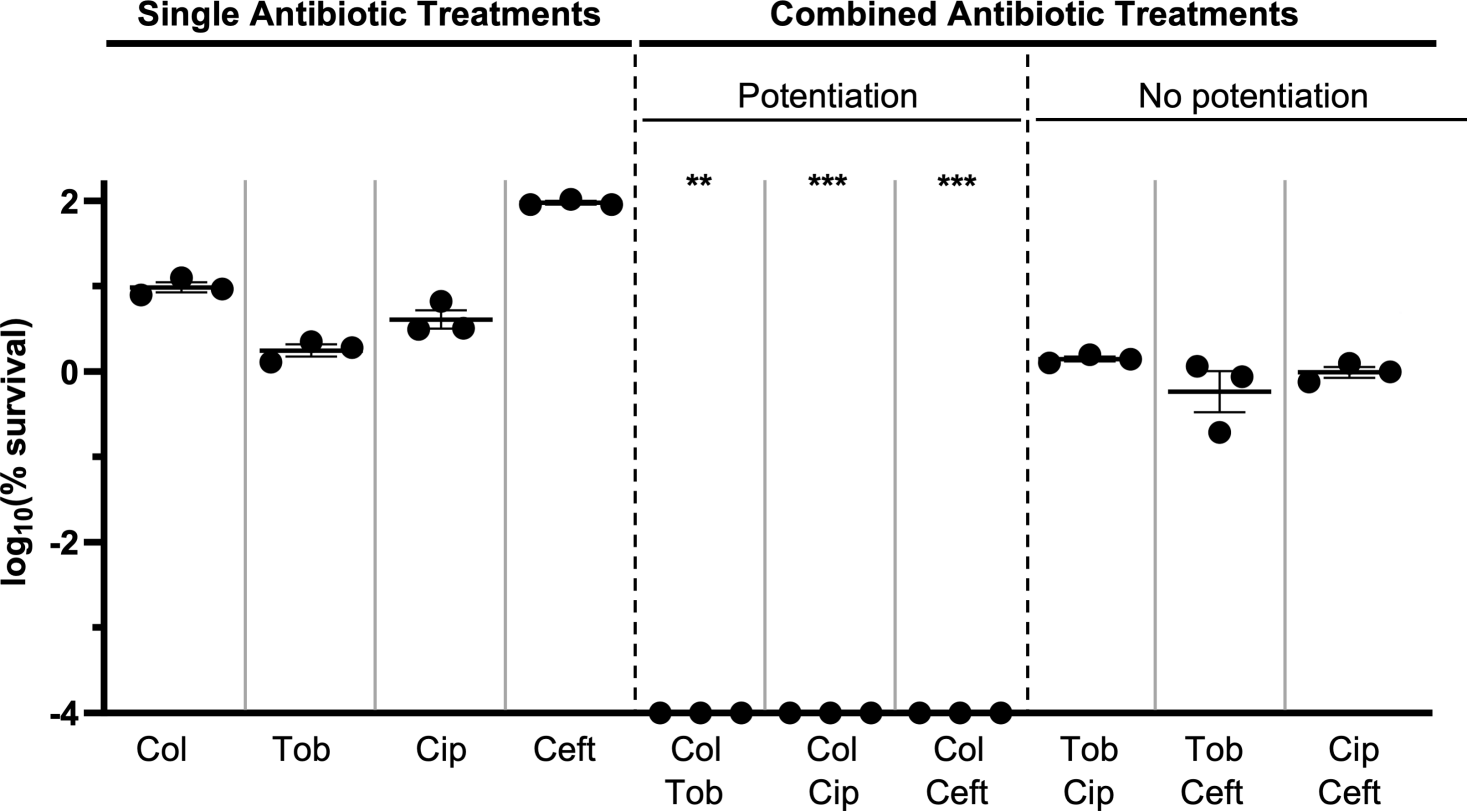
Most antibiotics do not interact with other classes of antibiotics to enhance the killing of *P. aeruginosa*. The log_10_(% survival) of WT *P. aeruginosa* cultures treated with colistin (Col), tobramycin (Tob), ciprofloxacin (Cip), ceftazidime (Ceft), or each antibiotic-antibiotic combination for 24 hours under hypoxic conditions. Final drug concentrations were 31.25 µg/mL tobramycin, 1 µg/mL ciprofloxacin, 15 µg/mL colistin, and 10 µg/mL ceftazidime. Compared to single-antibiotic treatments, only colistin addition enhances killing in combination with other classes of antibiotics. An antibiotic was defined as potentiating a second antibiotic when the amount of observed killing in the antibiotic-antibiotic combination was ≥1.5-log more than the predicted additive amount of killing, the latter of which was calculated by summing the observed log-killing values of both antibiotic-only treatments. All data points on the x-axis are below the detection limit. Data show the means of three replicates, and error bars show SEM. Statistical significance was determined by two-tailed Welch’s *t*-tests; ns = not significant, * = *P* < 0.05, ** = *P* < 0.01, *** = *P* < 0.001, and **** = *P* < 0.0001 comparing the predicted potentiation value (1.5-log killing more than the predicted additive amount of killing) to the observed amount of killing for colistin-antibiotic combinations.

Having observed chlorate’s ability to increase the lethality of different classes of antibiotics to kill *P. aeruginosa* under hypoxic conditions (Fig. 3A), we next sought to determine whether antibiotics themselves display a similar capacity. After treating hypoxic *P. aeruginosa* cultures (strain UCBPP-PA14) for 24 hours with different antibiotic-antibiotic combinations, we found that most classes of antibiotics do not result in potentiated killing (Fig. 7). Like chlorate, colistin addition also potentiates killing when combined with each of the other tested antibiotics (tobramycin, ciprofloxacin, and ceftazidime) to reduce *P. aeruginosa* viability to below our detection limit. However, the remaining three antibiotics did not exhibit potentiation in combination (Fig. 7). Thus, of the five drugs included in our study, only two (chlorate and colistin) displayed a capacity for wide-ranging potentiation with different classes of drugs.

## DISCUSSION

There is an intimate relationship between environmental conditions, bacterial physiology, and drug efficacy. Infection sites frequently harbor low O_2_ concentrations, causing many pathogens to grow slowly and adopt antibiotic-tolerant states and highlighting anaerobic bacterial metabolisms as promising targets for new therapies (23). It has been known for decades that chlorate is toxic to bacteria that respire nitrate via Nar because this enzyme reduces chlorate to generate highly toxic chlorite (Fig. 1A) (39, 40). In further support of chlorate’s therapeutic potential, we show that chlorate-antibiotic combinations overcome antibiotic-only treatment failure across varying O_2_ concentrations (Fig. 5). Chlorate promotes efficient killing across multiple *P. aeruginosa* isolates by potentiating different classes of antibiotics: aminoglycosides (tobramycin), fluoroquinolones (ciprofloxacin), beta-lactams (cephalosporins: ceftazidime), and polymyxins (colistin; Fig. 3 and 6). In the case of ceftazidime, chlorate addition reduced the toxic dose by >100-fold (Fig. 4). These findings underscore the potential for chlorate to enhance the efficacy of antibiotic treatments in environments that are typically characterized by antibiotic recalcitrance.

In previous work, we showed that 10 mM chlorate treatment improved wound healing outcomes in a *P. aeruginosa*-infected chronic wound diabetic mouse model (38). For those experiments, chlorate was administered topically to the wound. Thus, we envision chlorate treatment would be administered locally, such as either topically for delivery to a wound or inhaled for local delivery to the lungs (e.g., *P. aeruginosa* lung infection in people with CF). Of note, prior animal studies showed that high doses of ingested chlorate were well tolerated (41), recommending another potential route for treating pathogens in the gut. Encouragingly, many commensal bacteria, such as those found in the gut or on the skin, grow via fermentation rather than respiration (82, 83) and are thus less likely to be affected by chlorate treatment. Future studies will explore chlorate toxicity and chlorate-antibiotic interactions in other bacteria, including both commensals and pathogens, the latter of which are often facultative anaerobes known to utilize Nar-mediated nitrate respiration (26–38).

We also showed that chlorate and colistin synergize with different classes of antibiotics, whereas the remaining antibiotics in this study did not enhance killing in combination (Fig. 7). Most research examining antibiotic-antibiotic interactions in *P. aeruginosa* uses growth-based assays (e.g., checkerboard assay), which are not directly comparable to testing combination therapies under O_2_-limited, slow-growing conditions. Still, prior work with growth-based assays has also found that colistin frequently synergizes with other antibiotics, whereas ciprofloxacin and ceftazidime were less likely to exhibit synergy in combination with other drugs (84–86). Colistin, like chlorate, is more toxic to *P. aeruginosa* under O_2_-limited conditions compared to O_2_-replete conditions (87, 88). Further, colistin’s bactericidal activity was shown to be independent of reactive oxygen species (ROS) accumulation, specifically hydroxyl radical formation (89), whereas ROS generation is considered an important contributor to toxicity for numerous other antibiotics (90). If chlorate and colistin both have ROS-independent mechanisms of killing, this might lead them to synergize with ROS-generating antibiotics by causing multiple stressors in the cell, particularly under O_2_-limited conditions. However, additional studies are needed to more fully understand these mechanisms of drug synergy.

Our current understanding of drug interaction mechanisms is remarkably poor, highlighting a troubling knowledge gap in infectious disease research; we do not understand why some drug combinations are exceptionally lethal to bacteria, while others are not (91, 92). Without understanding the underlying mechanisms, our current approach for identifying novel synergistic drug pairings typically involves empirically testing each pairwise drug combination using resource-intensive high-throughput screens. Our own results highlight the challenging, unpredictable nature of synergistic drug interactions. For example, we found that hypoxic cultures of *P. aeruginosa* remain viable when treated with chlorate or high concentrations of ceftazidime (Fig. 4). While it might be tempting to conclude that these drugs have little to no effect on *P. aeruginosa*, this interpretation is undermined by our observation that combined chlorate-ceftazidime treatment is highly lethal (Fig. 3 and 4). More likely, chlorate and ceftazidime each exert stresses on the cell that are not captured by viability measurements. Concurrent with using expensive high-throughput screens to identify new drug combinations, future work should also examine underlying mechanisms of drug potentiation and drug synergy, which could guide the rational design of novel synergistic drug pairings (91, 93, 94).

Chlorate is a promising candidate for pursuing future studies to uncover mechanisms of drug potentiation because it enhances killing in combination with drugs that have different mechanisms of action. In prior work, we showed that chlorate treatment kills *P. aeruginosa* by causing widespread protein oxidation, particularly by damaging newly synthesized proteins (71). Thus, one possible explanation for chlorate’s impressive capacity for antibiotic potentiation is that it disrupts the cell’s translational response to antibiotic stress. Another potential explanation is that chlorate serves as an electron acceptor, such that chlorate reduction increases the cell’s metabolic activity, which is known to potentiate antibiotic therapy (95). While we cannot rule out the latter mechanism entirely, we note that the addition of O_2_ was insufficient to potentiate some antibiotics (e.g., Fig. 2, colistin and ceftazidime), so we expect chlorate’s role as a metabolism-stimulating electron acceptor to make limited contributions to synergy with at least some antibiotics. By studying mechanisms of chlorate-antibiotic or colistin-antibiotic killing, we can identify the types of cellular stresses that render *P. aeruginosa* highly susceptible to antibiotic therapy. Ultimately, defining the cellular stresses that drugs impose individually and in combination will begin to illuminate mechanisms of drug synergy, which will enhance our ability to find or predict powerful new drug combinations in the fight against antibiotic treatment failure.

## MATERIALS AND METHODS

### Bacterial strains and growth conditions

Strains used in this study include wild type (WT) *P. aeruginosa* UCBPP-PA14 and an isogenic strain with a markerless deletion of the *narGHJI* genes (referred to as *Δnar*) (70). Other *P. aeruginosa* strains used in this study include the common laboratory strain PAO1, a clinical isolate collected from a diabetic mouse model of chronic wounds (RPA [38]), and clinical isolates collected from people with CF including mucoid isolates (FRD1 [80], PA267, PA268, and PA269) and non-mucoid isolates (CF_early [81], PA266, and PA270). PA266–PA270 strains were isolated from people with CF at the Keck Hospital of USC. All strains were grown in Luria Broth (Miller’s LB Broth; Research Products International) and, where specified, supplemented with 40 mM potassium nitrate (KNO_3_; Sigma Aldrich) to stimulate Nar activity as nitrate respiration is required or important for *P. aeruginosa* growth under anoxic or hypoxic conditions, respectively. Oxic cultures were incubated at 37°C with shaking at 250 rpm. Hypoxic cultures were incubated at 37°C under static conditions. Anoxic cultures were incubated statically at 37°C in an anaerobic glove box with a 95% N_2_ and 5% H_2_ atmosphere.

### Drug treatment assays

Overnight cultures of *P. aeruginosa* were grown hypoxically for 24 hours in 10 mL of LB supplemented with 40 mM potassium nitrate; clinical isolate PA269 is slow growing, and thus, cultures were grown for 48 hours under the same conditions. Overnight cultures were pelleted and resuspended in fresh LB (lacking nitrate) at a high cell density (OD_500_ = 2). Next, 180 µL of resuspended culture was added to the well of a 96-well plate (Genesee Scientific) along with 20 µL of treatment (total volume per well = 200 µL). The 20 µL treatment volume consisted of 20 µL of sterile water for control conditions, 10 µL of sterile water and 10 µL of drug solution for single drug conditions, and 10 µL each of two different drug solutions for drug combination conditions. High-density cultures were incubated with or without drug(s) for 24 hours at 37°C under oxic, hypoxic, or anoxic conditions before plating for viable plate counts to determine percent survival. To limit evaporation, cultures exposed to drugs under hypoxic or anoxic conditions were incubated in humidified chambers. Under oxic conditions, evaporation was prevented by sealing the 96-well plate with micropore tape and filling empty wells with 200 µL of sterile water.

The following drug stock solutions (made in water) were added to wells of a 96-well plate in antibiotic treatment assays: 10 µL of 200 mM sodium chlorate (final = 10 mM), 10 µL of 625 µg/mL tobramycin (final = 31.25 µg/mL), 10 µL of 20 µg/mL ciprofloxacin (final = 1 µg/mL), 10 µL of 300 µg/mL colistin (final = 15 µg/mL), and 10 µL of 200 µg/mL ceftazidime (final = 10 µg/mL). Additional experiments were conducted using a range of ceftazidime concentrations that were achieved by adding 10 µL of a 20, 2,000, or 20,000 µg/mL stock solution to wells of a 96-well plate.  

### Viable plate counts for quantifying percent survival

Viable plate counts were determined for untreated or drug-treated samples by serially diluting samples in phosphate-buffered saline (PBS). Six 1:10 serial dilutions were made in PBS, and 10 µL of each dilution and of the undiluted culture were plated onto LB agar, allowing for viability quantification across seven orders of magnitude. LB plates were incubated for 24 hours at 37°C and then moved to the bench top to incubate for an additional 24 hours at room temperature to allow for the growth of slow-growing colonies; clinical isolate PA269 is slow growing, and thus, LB plates were incubated at 37°C for 48 hours before being moved to the bench top for additional incubation at room temperature. Colonies were counted to calculate CFU per mL for each sample. Percent survival was determined by dividing the CFU per milliliter value of each treated sample by the CFU per milliliter value of a control sample and multiplying by 100; the control sample value was the average of triplicate CFU per milliliter values of the culture at *t* = 0 (i.e., after washing and resuspending cultures to OD_500_ = 2 and just before the 24-hour drug incubation).

### MIC assay

The MIC of tobramycin, ciprofloxacin, colistin, and ceftazidime was determined for *P. aeruginosa* UCBPP-PA14 using the broth microdilution method. Overnight cultures were grown under hypoxic conditions in LB medium supplemented with 40 mM KNO_3_. Overnight cultures were pelleted and resuspended to a final cell density of 5 × 10^5^ CFU/mL in fresh LB. Cultures were incubated with twofold dilutions of each antibiotic for 24 hours at 37°C under static conditions. After drug incubation, the OD_500_ of each culture was measured to determine the lowest antibiotic concentration that inhibited growth.

## SUPPLEMENTAL MATERIAL

Supplemental material aem.01425-25-s0001.docx Table S1; Fig. S1.

ASM does not own the copyrights to Supplemental Material that may be linked to, or accessed through, an article. The authors have granted ASM a non-exclusive, world-wide license to publish the Supplemental Material files. Please contact the corresponding author directly for reuse.
