# Supplementary material for: Hijacking anaerobic metabolism to restore antibiotic efficacy in Pseudomonas aeruginosa
Source: Appl Environ Microbiol. 2025 Sep 29;91(10):e01425-25. doi: 10.1128/aem.01425-25 (PMC12542746; doi:10.1128/aem.01425-25)
Supplement: Supplemental material — Table S1; Fig. S1. [file aem.01425-25-s0001.docx]

**Supplemental Materials for**

**Hijacking anaerobic metabolism to restore antibiotic efficacy**

**in *Pseudomonas aeruginosa***

by

Zealon Gentry-Lear^1,2,*^, Celine Lopez Padilla^1,*^, and Melanie A. Spero^1,#^

^1^Institute of Molecular Biology, University of Oregon, Eugene, OR, USA

^2^Present address: Department of Microbiology, University of Washington, Seattle, WA, USA

^*^Authors contributed equally to this work

^#^Corresponding Author

University of Oregon

1229 University of Oregon

Eugene, OR 97403

Telephone: 541-346-1440

Email: mspero@uoregon.edu

**Supplementary Table S1:** Minimum inhibitory concentration (MIC) values of antibiotics used in this study for *Pseudomonas aeruginosa* UCBPP-PA14

| **Antibiotic** | **PA14 MIC  (µg/mL)** | **Interpretive categories and MIC breakpoints (µg/mL)^a^** | | |
| --- | --- | --- | --- | --- |
|  |  | **S** | **I** | **R** |
| Tobramycin | 2 | ≤ 1 | 2 | ≥ 4 |
| Ciprofloxacin | 0.125 | ≤ 0.5 | 1 | ≥ 2 |
| Colistin | 2 | - | 2 | ≥ 4 |
| Ceftazidime | 2 | ≤ 8 | 16 | ≥ 32 |

^a^All MIC breakpoints are from CLSI M100 ED35:2025

**Supplementary Figure 1: Nar is required for enhancing chlorate-ceftazidime killing under oxic conditions.** The log_10_(% survival) of **A.** WT or **B.** ∆*nar P. aeruginosa* cultures treated with chlorate (Chlor), ceftazidime (Ceft), or the combination for 24 hours incubated under oxic conditions. Final drug concentrations were 10 mM chlorate and 10 µg/mL ceftazidime. Data show the means of 3 replicates and error bars show standard error of the mean. Statistical significance was determined by two-tailed Welch’s t-tests; ns = not significant, * = p < 0.05, ** = p < 0.01, *** = p < 0.001, **** = p < 0.0001
